# Psychometric evaluation of PHQ–9 and GAD–7 among community health volunteers and nurses/midwives in Kenya following a nation-wide telephonic survey

**DOI:** 10.3389/fpsyt.2023.1123839

**Published:** 2023-05-30

**Authors:** Sabina Adhiambo Odero, Paul Mwangi, Rachel Odhiambo, Brenda Mumbua Nzioka, Constance Shumba, Eunice Ndirangu-Mugo, Amina Abubakar

**Affiliations:** ^1^Institute for Human Development, Aga Khan University, Nairobi, Kenya; ^2^School of Nursing and Midwifery, Aga Khan University, Nairobi, Kenya

**Keywords:** anxiety, depression, community health volunteers, PHQ-9, GAD-7, nurses/midwives, reliability, validity

## Abstract

**Background:**

Nurses/midwives and Community Health Volunteers (CHVs) are exposed to chronic stressors putting them at risk of developing mental health problems. This has been exacerbated by the COVID-19 pandemic. There is limited empirical evidence of the burden of mental health problems among health care workers partly due to the lack of adequately standardized and validated measures for use among health care workers in Sub-Saharan Africa. This study aimed to perform the psychometric evaluation of the PHQ-9 and GAD-7 administered to nurses/midwives and CHVs across 47 counties in Kenya.

**Methods:**

Between June and November 2021, a national survey on mental well-being and resilience among nurses/midwives and CHVs was conducted via telephone interviews. The survey had a total sample size of 1907 nurses/midwives and 2027 CHVs. Cronbach’s alpha and MacDonalds’ omega were used to evaluate the scale’s internal consistency. Confirmatory Factor Analysis (CFA) was used to test the one-factor structure of the scales. Multi-group CFA was applied to evaluate the generalizability of the scales across the Swahili and English versions, and among male and female health workers. The Spearman correlation was used to assess the tools’ divergent and convergent validity.

**Results:**

The internal consistency of PHQ-9 and GAD-7 was good, with alpha and omega values above 0.7 across study samples. CFA results indicated a one-factor structure of the PHQ-9 and GAD-7 for both nurses/midwives and CHVs. Multi-group CFA showed that both scales were unidimensional across both language and sex. The PHQ-9 and GAD-7 were positively correlated with perceived stress, burnout, and post-traumatic stress disorder, indicating convergent validity. The PHQ-9 and GAD-7 were significantly negatively correlated with resilience and work engagement, supporting divergent validity.

**Conclusion:**

The PHQ-9 and GAD-7 are unidimensional, reliable, and valid tools for screening depression and anxiety among nurses/midwives and CHVs. The tools can be administered in a similar population or study setting using either Swahili or English.

## 1. Introduction

Human Resources for Health (HRH) remains a key determinant of a well-functioning health system and is essential in improving population and individual health (1). However, with the global shortages in health workforce compared to population needs, health workers are continually exposed to work stressors, placing them at a high risk of psychological distress. These stressors are associated with high workload, long working hours, poor supply of required resources, poor working environments, among others (2). Kenya, like many other Low-and Middle-Income Countries (LMICs), also faces these health supply challenges and is dependent on nurses, midwives, and community health volunteers to meet the health services demands (3–5).

The ongoing COVID-19 pandemic has placed additional stressors on health workers, including fears and anxieties related to personal safety, possible infection and transmission to patients and family members (6). The health workers have experienced taxing working conditions with a relatively high influx of critically ill patients and death rates, as well as random redeployment of the clinical staff into unfamiliar territories to help contain the virus spread (7). They have also undergone loss or deterioration of personal support networks due to social stigmatization and isolation that risk eroding coping mechanisms, psychosocial wellbeing and resilience (8, 9).

Studies have shown that these stressors can have negative consequences on health workers’ mental health (10–14), commonly depressive symptoms and generalized anxiety (15). Depression and anxiety are usually associated as co-morbid conditions, with anxiety often contributing to occurrence of depression (16). Additionally, symptoms of psychological distress can appear as secondary presentations, including after the stressors end, and with ranging severity (17). Health workers who are experiencing psychological distress provide poor quality care to their patients due to impaired performance, reduced productivity, and increased turnover (2, 18, 19). For example, previous studies have shown frequent absenteeism and high turnover among nurses experiencing anxiety (20–23).

In Kenya, studies conducted during the COVID-19 pandemic reported that health workers were experiencing mental health problems such as depression, anxiety, insomnia and stress. Kwobah and colleagues reported 36% of HCWs experienced anxiety, 32.1% depression, 24.2% insomnia and 64.7% post-traumatic stress disorder in their online survey of mental health disorders among Kenyan health care workers in the early phase of COVID-19 (24). Another study conducted in Kenya across three different hospitals during the COVID-19 pandemic showed that health care workers experienced depression (53.6%), anxiety (44.3%), insomnia (41.1%), distress (31.0%), burnout (45.8%) (25). Additionally, a survey among nurses working in a tertiary health facility in Kenya during COVID-19 reported depression, anxiety, insomnia, distress and burnout at 45.9, 48.2, 37.0, 28.8, and 47.9% (26). The risk factors for depression and anxiety were higher among females health workers, less than 30 years of age, not being married and those with less than 10 years of work experience (27). However, none of these studies reported the reliability or validity of the measures they employed to assess the mental health of the healthcare workers. It is crucial to ascertain that the measures used in assessing healthcare workers’ mental health are appropriate, given the high levels of reported mental health issues. This is because having reliable and valid tools is an essential initial step in ensuring that healthcare workers receive the necessary mental health support they need.

Community health volunteers (CHVs) in Kenya play an integral role in health systems providing linkages between the community and health system and complementing the shortages in health workers in providing community-based health services. During the COVID-19 pandemic they have been providing services such as contact tracing, isolation and mobilization (28). Their potential and ability to provide mental health support to communities has been documented in previous studies (29–33), however, little has been done to document how they themselves receive psychosocial support. A recent survey by the George Institute and the Health Systems Global Thematic Working Group on Community Health Workers found that the mental health support received by CHWs during the COVID-19 was offered by implementing partners (34). The survey aimed at assessing availability of mental health support for CHWs during the COVID-19 pandemic. Reports from survey participants showed that CHWs were experiencing mental distress and showing symptoms of anxiety, depression, increased stress, and complaints of high workload and burnout (34).

The commonly used tools to measure anxiety and depression are the 9-item Patient Health Questionnaire (PHQ-9) and the 7-item Generalized Anxiety Disorder (GAD-7), respectively. PHQ-9 has been used among different populations in Kenya including adolescents and people living with HIV (35–37), while GAD-7 has been used among adolescents and adults living with HIV, and caregivers of children in community based early child development program (38–40).

However, as Jaguga and Kwobah (41) found in their review, Kenya’s mental health response is still lacking, with an unmet need for psychological support and mental health surveillance system. This highlights a need to validate the existing measures for mental health disorders among different groups in Kenya. Validation is important to check content, conceptual, semantic and idiomatic equivalence (42), ensuring use of contextually appropriate and locally validated mental health measures to contribute meaningful epidemiological data in depression and anxiety in the country, and globally. To the best of our knowledge, these measures have not been validated among healthcare workers in Kenya.

This study, therefore, set out to assess the validity of the PHQ-9 and GAD-7, both in English and a locally translated version to Swahili, among nurses, midwives, and CHVs. Swahili is a national language in Kenya and is easily understood by majority of the population alongside English. To the best of our knowledge, no other study has validated the English and Swahili versions of GAD-7 and PHQ-9 among nurses, midwives, and CHVs in Kenya. We specifically report on internal consistency, convergent validity, divergent validity, construct validity, and measurement of invariance across languages.

## 2. Materials and methods

### 2.1. Study site and population

This study was conducted across the 47 counties in Kenya. Each of the 47 counties has a semi-autonomous public health system following devolution by the government in 2013. County governments oversee delivery of healthcare services while the national government retained the policy and regulatory functions (43, 44).

This analysis is part of a larger cross-sectional study, whose aim was to generate evidence on the mental wellbeing and resilience of nurses, midwives, and community health volunteers (CHVs) in Kenya, in the context of COVID-19. It was conducted among health workers who had worked for 6 months or longer, spoke English or Swahili, and who consented to participate in the study.

### 2.2. Sample and data collection procedures

#### 2.2.1. Sample size

We used STATA/SE 14.1 to compute the sample size (45) using the formula


$$n={\left(Z_{1-\frac{\alpha }{2}}\right)}^{2}p\left(1-p\right)/d^{2}$$

where,

*n* is the sample size,

*Z* is the statistic for the level of confidence,

*p* is the prevalence, and.

*d* is the margin error.

We estimated the prevalence of mental health problems among health care workers to be 24.73% based on a survey conducted in Italy 2020 (46). We used a 95% confidence level, a 2% margin of error and obtained a sample size of 1788. After accounting for a 10% non-response rate, a sample of 1900 nurses and midwives and 1900 CHVs was sufficient.

#### 2.2.2. Recruitment of nurses and midwives

There is a national registry of all registered nurses and midwives in Kenya held by the Nursing Council of Kenya (NCK). We used this existing database to apportion the sample size (n = 1900) to each county using proportionate to size sampling (47). In this way, the sample size per county was proportional to the total number of nurses and midwives that the county had based on the NCK register at that point in time. NCK could not provide us with the personal information we needed (names, phone numbers, email addresses) to contact the nurses/midwives due to the Data Protection Act, 2019. Therefore, we sent a text message to all the nurses/midwives in the NCK database at the time (*N* = 45,942) inviting them to participate in the study; we received responses from 4,547 nurses and midwives, out of whom 4,377 consented to being contacted to participate in the study. As such, consent to participate in the study came from the participants themselves, not the NCK. The study team followed up with those who had consented to be contacted via phone calls to schedule the interview. At the scheduled time, a Field Enumerator (FE), called and conducted the interview.

Out of those who responded and expressed their willingness to participate in the study, individual nurses and midwives were recruited into the study through sampling by replacement while maintaining the aspect of proportionality to the number of nurses and midwives in each county. In cases where the selected nurse or midwife was missing or had changed their mind about participating or was not available, another nurse was randomly selected from the remaining pool of nurses in the county.

#### 2.2.3. Recruitment of community health volunteers

There was no comprehensive register of all CHVs in the country available to us, therefore, we distributed the target number of CHVs (*n* = 1900) proportionately across all the 47 counties, such that we targeted to interview an equal number of CHVs in each county (41 CHVs). Approval to get in touch with the CHVs was sought from each county, and the County Community Health Focal Persons helped us disseminate information about the study through the sub-county focal persons and the community health assistants. The contact details of CHVs who were willing to participate were forwarded to the study team who went ahead to call, schedule, and conduct interviews.

#### 2.2.4. Data collection

Data collection was carried out via telephone calls. During the interviews, the study information sheet was read out by trained research assistants to the eligible participants. They shared information about the project, risks and benefits to participants, details about what to expect if they agreed to participate and the project’s contact information. All potential informants were given the chance to decline participation if they wanted to since participation was voluntary. The interviewer also sought consent to audio record the telephonic interviews. Once verbal consent was obtained, the interviewer signed a copy of the consent form as proof and an acknowledgement that they followed standard procedures of obtaining informed consent.

### 2.3. Measures

#### 2.3.1. Sociodemographic tool

The sociodemographic questionnaire captured data on age, level of education, marital status, religiosity and social support, years of experience, type and level of health facility, number of working hours, salary receipt on time, regularity of receiving salary, availability of health insurance.

#### 2.3.2. Mental health measures

The following tools were used to assess levels of mental distress among the nurses, midwives, and community health workers.

#### 2.3.3. Patient health questionnaire-9

PHQ-9 was used to assess depressive symptoms. It comprises a Likert scale of “0” (not at all) to “3” (nearly every day) applied to the 9 items in the measure. Respondents were asked how often they had been bothered by each of the symptoms over the previous 2 weeks, and a score ranging from 0 to 27 derived from summation of their responses. A cut-off score of ≥10 was used to define a positive screen for depressive symptoms, which is consistent with previous studies from Kenya (35–37).

#### 2.3.4. Generalized anxiety disorder-7

GAD-7 was used to assess symptoms of generalized anxiety over the previous 2 weeks in this study. GAD-7 is a seven-item screening measure, scored on a four-point Likert scale ranging from zero (not at all) to three (nearly every day). Scores of 5–9, 10–14, 15–19, and 20–27 were used to indicate mild, moderate, moderately severe, and severe levels of generalized anxiety, respectively, consistent with other studies from Kenya (38–40).

#### 2.3.5. Perceived stress scale-10

The PSS-10 is used to assess current stress levels among a study population. The field team read out to health workers in this study a list of 10 symptoms and asked them to rate their stress levels based on a scale of zero to four. Some of the items were reverse coded during analysis. Total score ranged from 0 to 40, with score higher than 27 indicating high perceived stress. The PSS-10 has been used in Kenya among maternity health care providers (48) and medical students (26), pregnant women with adverse childhood experiences (49), among other populations, but to our knowledge has not been validated.

#### 2.3.6. Primary care PTSD screen for DSM-5

This is a 5-item tool designed to assess probable PTSD in primary care settings. The measure first asks a single question on exposure to traumatic events, if the respondent answers with a “no” then the assessment is complete with a score of zero. If they respond with a “yes” then the respondent goes ahead to respond to the five items in the measure, scored dichotomously as zero or one (0 = No; 1 = Yes). Total scores range from 0 to 5. Those who responded positively to 3 out of 5 were considered to have probable PTSD, which is the recommended cut-off (50).

#### 2.3.7. Brief resilience scale

The Brief Resilience Scale (BRS) scale is used to assess how one can adapt and bounce back when they experience stressful events. The Brief Resilience Scale (BRS) has been used among adolescents, young adults, and adults from different settings and has shown good validity and reliability across different languages (51–55).

#### 2.3.8. Oldenburg burnout inventory

Burnout has been measured extensively among students and employees using different measures, including the Oldenburg Burnout Inventory (56). The measure has a series of 16 statements: 8 measuring disengagement and 8 measuring exhaustion. The respondents were asked to rate the items on a 4-point Likert scale indicating their level of agreement or disagreement with the items. The scores were then summed up on a scale ranging from 16 to 64.

#### 2.3.9. Utrecht work engagement scale – 9

The UWES-9 was used to assess health workers’ engagement in work. UWES-9 is a nine-item screening measure, scored on a 7-point Likert scale ranging from zero (never) to 6 (always). The measure has three subscales of vigor, dedication, and absorption. The measure has been used among rescue workers in Portugal (57), community health workers in Sierra Leone (58), and nurses in Vietnam (59) all showing good validity and reliability. However, more work needs to be done to assess validity and reliability of the UWES-9 in Sub Saharan Africa (SSA).

### 2.4. Data analysis

The data were analyzed using R statistical software version 4.1.2 (60). The data sets for CHVs and nurses/midwives were analyzed separately. The sociodemographic variables (sex, age, level of education, marital status, psychosocial support from religion, work duration, type of health facility, working hours a day, monthly income, and health insurance) were summarized using descriptive statistics. The frequency and proportion were used for categorical variables, while the mean and standard deviation were used for continuous variables.

The internal consistency of PHQ-9 and GAD-7 was computed using Cronbach’s alpha (α) and Macdonald’s omega (ω) (61). The scales show good internal consistency if the values of Cronbach’s α and Macdonald’s ω are above 0.7 (62). Additionally, the item’s score contributing to the scale was assessed using corrected item-total correlations (CITCS) and a value greater than 0.4 indicated the item had been homogeneous in measuring the scale (63). Convergent validity was assessed by correlating the PHQ-9 and GAD-7 total scores, respectively, with the Perceived Stress Scale-10 (PSS-10) total score, Oldenburg Burnout Inventory (OBI) total score, and posttraumatic stress disorder total score. Divergent validity was evaluated by correlating the PHQ-9 and GAD-7 total scores, respectively, with the Brief Resilience Scale (BRS) total score, Utrecht Work Engagement Scale-9 (UWES-9) total score. Spearman correlation coefficients were used for both convergent and divergent validity. The correlation coefficients values of <0.3, 0.3 to 0.5, and above 0.5, indicated weak, moderate, and strong correlation, respectively.

The Kaiser-Meyer-Olkin (KMO) Test for sampling adequacy was used to assess whether the data sets were appropriate for factor analysis, and a value of KMO estimate above 0.7 was acceptable (64). The relationship between the items was assessed using Bartlett’s test of sphericity. The construct validity of PHQ-9 and GAD-9 was assessed using confirmatory factor analysis (CFA) (65). The CFA model goodness of fit was assessed using the fit indices; the Root Mean Error of Approximation (RMSEA) < 0.08 acceptable fit and < 0.05 good fit, the Standardized Root Mean Square Residual (SRMR) < 0.06, and the Comparative Fit Index (CFI) and the Tucker Lewis Index (TLI) > 0.95 indicating excellent fit (66).

Measurement of invariance was used to evaluate whether the PHQ-9 and GAD-7 had an invariant one factor across the languages (Kiswahili, English and both) and across sex (males vs. females). Note, the data sets from CHVs and nurses were combined for the measurement invariance analysis. In this analysis, a sequence of invariance models was tested; a configural invariance model, metric invariance model, and scalar invariance model. The invariance models were contrasted, metric versus configural and scalar versus metric using CFI, and the ∆CFI ≤ 0.01 indicated unidimensionality of the PHQ-9 factor and GAD-7 factor across the languages. For all results, a 5% level of significance was used.

### 2.5. Ethical considerations

The study received approval from Aga Khan University’s Institutional Ethics Review Committee (IERC) for Kenya (IERC number 2021/IERC-32) and the National Commission for Science, Technology and Innovation (NACOSTI/P/21/10034). All study participants provided verbal consent captured in the audio recording of the interview, since data collection was conducted via telephonic interviews.

## 3. Results

### 3.1. Participants sample characteristics

The study comprised a total sample of 2027 CHVs and 1907 nurses/midwives. The participants’ socio-demographic and psychosocial characteristics are shown in Table 1. The mean age of CHVs was 43.93 (SD = 11.05), most were females (52.8%), and 44.5% had secondary education or higher. More than half of CHVs were married (79.0%), received psychosocial support from religion (53.9%), had worked for less than 10 years (68.5%), and were not receiving a salary (90.2%). The mean age for nurses/midwives was 34.12 (SD = 10.28), most nurses were females (59.9%), and 19.9% had a bachelor’s degree in nursing or higher. Most nurses worked in public health facilities (53.9%), had work experience of fewer than 10 years (72.0%), and worked 8 to 11 h a day (64.9%). Of the total samples of nurses, 27.5% did not receive psychosocial support from religion, and 8.6% had no health insurance.

**Table 1:** Participants’ sociodemographic psychosocial characteristics.

	Community health workers	Nurses and midwives
	*n* (%)	*n* (%)
Over-all	2027	1907
Sex		
Female	1,070 (52.8)	1,143 (59.9)
Male	957 (47.2)	764 (40.1)
Age *means (SD)*	43.93 ± 11.05	34.12 ± 10.28
Education level		
Secondary school	896 (44.2)	–
Nursing/midwifery (certificate)	3 (0.1)	73 (3.8)
Nursing/midwifery (Diploma)	5 (0.2)	1,381 (72.4)
BSc nursing	–	346 (18.1)
MSc nursing	–	35 (1.8)
Other	1,123 (55.4)	72 (3.8)
Marital status		
Single	213 (10.5)	774 (40.6)
Married	1,601 (79.0)	1,082 (56.7)
Divorced/Separated	62 (3.1)	28 (1.5)
Widowed/Widower	151 (7.4)	23 (1.2)
Psychosocial support from religion		
Yes	1,092 (53.9)	1,290 (67.6)
No	775 (38.2)	524 (27.5)
*Missing*	160 (7.9)	93 (4.9)
Work duration		
<10 years	1,388 (68.5)	1,373 (72)
11–20 years	557 (27.5)	263 (13.8)
21–30 years	53 (2.6)	175 (9.2)
31–40 years	7 (0.3)	63 (3.3)
>40 years	5 (0.2)	5 (0.3)
*Missing*	17 (0.8)	28 (1.5)
Health facility management		
Public	–	1,028 (53.9)
Private	–	689 (36.1)
Faith based organization	–	145 (7.6)
Others	–	45 (2.4)
Working hours/day		
<8	1883 (92.9)	195 (10.2)
8–11	112 (5.5)	1,237 (64.9)
12–16	23 (1.1)	453 (23.8)
>16	9 (0.4)	22 (1.2)
Receive salary		
Every month	138 (6.8)	1829 (95.9)
Every 3 months	19 (0.9)	9 (0.5)
Every 2 months	5 (0.2)	1 (0.1)
Unknown (no established pattern)	33 (1.6)	25 (1.3)
Other	3 (0.1)	33 (1.7)
*Missing*	1829 (90.2)	10 (0.5)
Receive salary on time		
Yes	48 (2.4)	1,002 (52.5)
No	99 (4.9)	473 (24.8)
Sometimes	51 (2.5)	422 (22.1)
*Missing*	1829 (90.2)	10 (0.5)
Health insurance		
Yes	785 (38.7)	1743 (91.4)
No	1,242 (61.3)	164 (8.6)

### 3.2. Reliability

The results of internal consistency are depicted in Table 2. The results revealed that the PHQ-9 had a good internal consistency with Cronbach’s α and MacDonald’s ω values above 0.7 for CHVs, and nurses/midwives, respectively. Similarly, the GAD-7 had a good internal consistency with α and ω values above 0.7 for both groups (Table 2). The item test corrected correlation of GAD-7 ranged from 0.66–0.73 for CHVs and 0.64–0.72 for nurses/midwives, while PHQ-9 ranged from 0.53–0.67 for CHVs and 0.53–0.67 for nurse/midwives. These results indicate that all the items had a good contribution in measuring PHQ-9 scale and GAD-7 scale, respectively, since all the item corrected correlations were above 0.4. Further, the results revealed that if the item was deleted, the alphas values were not greater than the overall alpha of the PHQ-9 and GAD-7, respectively.

**Table 2:** Internal consistency for GAD-7 and PHQ-9.

	Cronbach’s alpha (95% CI)	MacDonald’s omega (95% CI)
*Community health workers*		
PHQ-9	0.78 (0.77–0.80)	0.77 (0.76–0.80)
GAD-7	0.82 (0.81–0.84)	0.82 (0.80–0.84)
*Nurses and midwives*		
PHQ-9	0.74 (0.73–0.76)	0.75 (0.73–0.77)
GAD-7	0.80 (0.81–0.82)	0.81 (0.80–0.83)

### 3.3. Convergent validity

Table 3 summarizes the divergent and convergent results. The PHQ-9 was strongly correlated with GAD-7 (*r* = 0.75, *p* < 0.001) in CHVs and (*r* = 0.75, *p* < 0.001) in nurses/midwives. PHQ-9 had a significant and moderate positive correlation with PSS-10 (*r* = 0.41, *p* < 0.001) for CHVs, and PSS-10 (*r* = 0.47, *p* < 0.001), PTSD (*r* = 0.32, *p* < 0.001), and OBI (*r* = 0.41, *p* < 0.001) for nurses/midwives. There was also a significant and moderate positive correlation between GAD-7 and PSS-10 (*r* = 0.36, *p* < 0.001), PTSD (*r* = 0.30, *p* < 0.001) for CHVs and PSS-10 (*r* = 0.48, *p* < 0.001), OBI (*r* = 0.41, *p* < 0.001), and PTSD (*r* = 0.32, *p* < 0.001) for nurse/midwives. Furthermore, the PHQ-9 had a significant and weak correlation with OBI (*r* = 0.26, *p* < 0.001) and PTSD (*r* = 0.26, *p* < 0.001) for CHVs.

**Table 3:** Divergent and convergent validity of PHQ-9 and GAD-7.

	Community Health workers		Nurses and midwives	
	PHQ-9	GAD-7	PHQ-9	GAD-7
*Convergent validity*				
Patient Health Questionnaire (PHQ-9)	1	0.75*	1	0.75*
Generalized Anxiety Disorder (GAD-7)	0.75*	1	0.75*	1
Perceived Stress Scale-10 (PSS-10)	0.41*	0.36*	0.47*	0.48*
Oldenburg Burnout Inventory (OLBI)	0.26*	0.29*	0.41*	0.41*
Posttraumatic stress disorder (PTSD)	0.26*	0.30*	0.32*	0.31*
*Divergent validity*				
Brief Resilience Scale (BRS)	−0.19*	−0.19*	−0.29*	−0.30*
Utrecht Work Engagement Scale (UWES) – 9	−0.10*	−0.14*	−0.15*	−0.16*

*p value < 0.05.

### 3.4. Divergent validity

The results revealed a significant and weak negative correlation for PHQ-9 with BRS (*r* = −0.19, *p* < 0.001), UWES (*r* = −0.10, *p* = <0.001) for CHVs, and BRS (*r* = −0.29, p < 0.001), and UWES (*r* = −0.15, *p* < 0.001) for nurses/midwives. Also, there was a significant and weak negative correlation for GAD-7 with BRS (*r* = −0.19, *p* < 0.001), and UWES (*r* = −0.14, *p* < 0.001) for CHVs, and BRS (*r* = −0.30, *p* < 0.001), and UWES (*r* = −0.16, *p* < 0.001) for nurses/midwives.

### 3.5. Construct validity

Confirmatory factor analysis (CFA) was employed to assess the unidimensionality of PHQ-9 and GAD-7. Before performing the CFA, the Kaiser-Meyer-Olkin measure and Bartlett’s test of sphericity were used to assess the data sets’ suitability for factor analysis. The findings revealed a KMO estimate value of 0.89 for PHQ-9 (CHVs), 0.89 for GAD-7 (CHVs), 0.86 for PHQ-9 (nurses/midwives), and 0.88 for GAD-7 (nurses/midwives), as well as a significant Bartlett’s test result (*p* < 0.001) for PHQ-9 and GAD-7 in both groups. These findings suggest that the data sets were adequate for factor analysis.

Table 4 summarizes the confirmatory analysis results for PHQ-9 and GAD-7. The findings revealed that the PHQ-9 and GAD-7 had a good one factor structure for CHVs and nurses/midwives, respectively, with all goodness of fit indices falling within the recommended thresholds. The factor loadings were all significant and ranged from 0.42 to 0.61 for PHQ-9 (CHVs), 0.58 to 0.69 for GAD-7 (CHVs), 0.39 to 0.56 for PHQ-9 (nurses/midwives), and 0.58 to 0.65 for GAD-7 (nurses/midwives). The factor loadings’ results were greater than the recommended threshold value of 0.35, indicating that the PHQ-9 and GAD-7 factors explained each item well, respectively.

**Table 4:** Confirmatory Factor Analysis for PHQ-9 and GAD-7.

Fit Indices	Chi-square statistic	RMSEA	SRMR	TLI	CFI
*Community health workers*					
PHQ-9	*χ*^2^ (5, *n* = 2027) = 13.24, *p* = 0.021	0.000	0.019	1.001	1.000
GAD-7	*χ*^2^ (14, *n* = 2027) = 26.34, *p* = 0.023	0.021	0.027	0.996	0.997
*Nurses and midwives*					
PHQ-9	*χ*^2^ (27, *n* = 1907) = 52.67, *p* = 0.002	0.022	0.030	0.989	0.992
GAD-7	*χ*^2^ (14, *n* = 1907) = 28.71, *p* < 0.001	0.013	0.023	0.998	0.999

### 3.6. Measurement invariance across languages (Kiswahili, English, and both) and across sex (males vs. females)

The results of the measurement of invariance are shown in Table 5. The model’s results where intercepts, factor loadings, and variances were set to be free but had the same factor and number of items across the two groups, language and sex, (configural invariance model) indicated the PHQ-9 and GAD-7 factors fitted the data well, respectively. Additionally, constraining all items to load equally across the two groups (metric invariance model) revealed that the PHQ-9 and GAD-7 factors had a good fit, respectively. Further, constraining the intercepts to be equal across two groups (scalar invariance model), the results showed that the PHQ-9 and GAD-7 factors had an excellent fit. The change of CFI (∆CFI) was used to contrast the sequential invariance models. The results indicated that comparing the metric invariance model versus the configural invariance model, the ∆CFI was 0.003 (languages) and < 0.001 (sex) for PHQ-9 and 0.002 (languages) and 0.001 (sex) for GAD-7.For scalar invariance model versus metric invariance model, the ∆CFI was 0.019 (languages) and 0.003 (sex) for PHQ-9 and 0.011 (languages) and 0.001 (sex) for GAD-7. The ∆CFI results for all model comparisons were less than 0.02, indicating that the PHQ-9 and GAD-7 scales had an invariant factor structure across the languages and sex, respectively.

**Table 5:** Measurement of invariance across Kiswahili vs. English tool versions and across males vs. females (for both CHVs and nurses/midwives).

	Fit Indices	PHQ-9	GAD-7
Confirmatory Factory Analysis	Chi-square statistic	*χ*^2^ (27, *n* = 3,930) = 59.35, *p* < 0.001	*χ*^2^ (14, *n* = 3,930) = 38.15, *p* < 0.001
	RMSEA (90% CI)	0.017 (0.011–0.024)	0.021 (0.021–0.013)
	SRMR	0.022	0.024
	TLI	0.994	0.996
	CFI	0.996	0.997
Tool version (Swahili vs. English)			
Configural invariance	Chi-square statistic	*χ*^2^ (81, *n* = 3,930) = 95.91, *p* = 0.123	*χ*^2^ (42, *n* = 3,930) = 46.65, *p* = 0.287
	RMSEA (90% CI)	0.012 (0.000–0.020)	0.009 (0.000–0.022)
	SRMR	0.025	0.023
	TLI	0.997	0.999
	CFI	0.998	0.999
Metric invariance	Chi-square statistic	*χ*^2^ (97, *n* = 3,930) = 131.35, *p* = 0.012	*χ*^2^ (54, *n* = 3,930) = 76.98, *p* = 0.022
	RMSEA (90% CI)	0.016 (0.008–0.023)	0.018 (0.007–0.027)
	SRMR	0.029	0.029
	TLI	0.995	0.997
	CFI	0.995	0.997
	∆CFI	0.003	0.002
Scalar invariance	Chi-square statistic	*χ*^2^ (113, *n* = 3,930) = 276.73, *p* < 0.001	*χ*^2^ (16, *n* = 3,930) = 175.88, *p* < 0.001
	RMSEA (90% CI)	0.033 (0.028–0.038)	0.036 (0.029–0.042)
	SRMR	0.038	0.038
	TLI	0.979	0.987
	CFI	0.978	0.986
	∆CFI	0.017	0.013
Sex (males vs. females)			
Configural invariance	Chi-square statistic	*χ*^2^ (54, *n* = 3,930) = 73.17, *p* = 0.042	*χ*^2^ (28, *n* = 3,930) = 45.65, *p* = 0.019
	RMSEA (90% CI)	0.013 (0.003–0.021)	0.018 (0.007–0.027)
	SRMR	0.022	0.023
	TLI	0.997	0.997
	CFI	0.997	0.998
Metric invariance	Chi-square statistic	*χ*^2^ (62, *n* = 3,930) = 83.50, *p* = 0.036	*χ*^2^ (34, n = 3,930) = 59.04, *p* = 0.005
	RMSEA (90% CI)	0.013 (0.004–0.020)	0.019
	SRMR	0.024	0.026
	TLI	0.997	0.996
	CFI	0.997	0.997
	∆CFI	<0.001	0.001
Scalar invariance	Chi-square statistic	*χ*^2^ (70, *n* = 3,930) = 113.13, *p* = 0.001	*χ*^2^ (40, *n* = 3,930) = 75.94, *p* = 0.001
	RMSEA (90% CI)	0.018 (0.011–0.024)	0.021
	SRMR	0.027	0.029
	TLI	0.994	0.995
	CFI	0.994	0.996
	∆CFI	0.003	0.001

## 4. Discussion

### 4.1. Summary of current study findings

Studies validating tools to assess depression or anxiety in SSA among health workers are limited. This study aimed to estimate the reliability and validity of the Swahili and English versions of the PHQ-9 and GAD-7 in measuring depression and generalized anxiety among community health volunteers and nurses/midwives in Kenya. The results show that the PHQ-9 and GAD-7 had excellent internal consistency in both CHVs and nurses/midwives. The factor structure of PHQ-9 and GAD-7 had significant factor loadings above 0.35 and acceptable fit indices, indicating depression and generalized anxiety constructs are valid in CHVs’ and nurses’/midwives’ populations, respectively. Additionally, the measurement invariance results revealed that the unidimensionality of PHQ-9 and GAD-7 was constant across the languages (Kiswahili, English and both) and across sex (males vs. females), respectively. These findings indicate that the PHQ-9 and GAD-7 are reliable and valid tools for assessing depression and generalized anxiety among CHVs and nurses/midwives.

### 4.2. Internal consistency

The internal consistency results of PHQ-9 were excellent, with Cronbach’s α and MacDonald’s ω values above 0.7 in both groups, and this supported the usefulness and reliability of PHQ-9. These findings are comparable with the results from previous studies in which alpha values were above 0.7 when PHQ-9 was validated in a similar working environment, 0.89 among medical students in Omani (67) and 0.80 among primary care attendants in Botswana (68). Similarly, the PHQ-9 was found to be reliable in other populations, 0.84 among both HIV-infected and uninfected populations in Kenya (37), 0.85 among patients with heart failure (69), and 0.87 among psychiatric patients in the United States (70). Consequently, the results of the study revealed good internal consistency of GAD-7 in both groups, which confirms results from past studies, 0.89 in the general population in Germany (71), 0.82 in the HIV population in Kenya (39), and 0.88 among patients in Portuguese (72).

### 4.3. Divergent and convergent validity

The significant and positive strong correlation between the depression and anxiety scores provided evidence of convergent validity, and these results concur with other studies’ findings between anxiety and depression (73, 74). Both depression and anxiety scores were positively correlated with burnout among nurses, which is consistent with findings from a study that sought to assess the relationship between coping styles and burnout and mental health among medical practitioners (75). The results also showed that perceived stress was correlated with depression and anxiety. The results obtained by Gorgich et al. showed that the high level of stress among nurses increases mental health problems (76). Also, other studies have shown a significant positive relationship between stress and mental health problems among health workers (77, 78). Additionally, depression and anxiety were positively related to a post-traumatic stress disorder, which affirms the finding of a study conducted in Istanbul among healthcare workers, which showed that PTSD was associated with a high level of depression and anxiety (79). For example, health workers working in high-risk environments are at risk of psychological distress, which can repeatedly occur, resulting in trauma that can lead to mental health problems (80, 81).

On the other hand, our findings showed that a high level of resilience was negatively associated with depression and anxiety. Gao et al. reported that nurses with higher resilience were less likely to experience mental health problems (82). Consequently, the results showed that the Utrecht Work Engagement Scale score was negatively related to depression and anxiety, and this is because health workers who work with enthusiasm and commitment are more likely to persevere in the face of adversity, which has been linked to lower mental health problems (83).

### 4.4. Construct validity

The CFA results from this study demonstrated that the PHQ-9 and GAD-7 scales are unidimensional in measuring the depression and anxiety constructs, respectively. Further, the measurement of invariance (configural, metric, and scalar) results across the languages (i.e., English, Kiswahili, or both) and sex (males vs. females) indicated that the unidimensional of PHQ-9 and GAD-7 scale was invariant across languages and sex, respectively. These findings suggest that depression or anxiety among male or female CHVs or nurses/midwives can be assessed through English, Kiswahili, or both languages using PHQ-9 and GAD-7 scales, respectively. Also, based on the finding of one structure of PHQ-9 and GAD-7 across nurses/midwives and CHVs indicates that depression and anxiety manifest the same way across the two populations. The one-factor structure of PHQ-9 is comparable with other previous studies conducted in the US among a diverse college population (African American, Asian American, European, American, Latino/American) (84), among HIV-affected and community controls populations in Kenya (37), and among outpatients departments in the major referral hospital in Ethiopia (85). Consequently, our results coincide with international studies of the one structure of the GAD-7 (71, 86, 87). We did not find another study that had validated PHQ-9 and GAD-7 among health workers, and we hope that these results provide a basis for further work to ensure there is a tool kit that can be used to evaluate the psychosocial wellbeing and mental health of health care workers.

## 5. Strengths and limitations

The study’s main strength was the large enough sample size for psychometric analysis, which resulted in reliable results. Furthermore, this is the first study to assess the validity of mental health tools among nurses/midwives and CHVs from all 47 counties in Kenya, ensuring a representative sample of this population. The tools were administered in both Swahili and English, which improved data quality by allowing participants to select either language. Finally, the availability of tools for conducting both convergent and divergent validity strengthens our findings by contrasting them with GAD-7 and PHQ-9. However, the study had some limitations. First, the sensitivity and specificity analysis were not reported because the study lacked a gold standard tool for assessing depression or anxiety. Second, no test–retest data were collected due to the busy schedule of health workers. Finally, we may have had selection bias since we only interviewed nurses/midwives who opted into the study, and had no data to compare whether their characteristics differed significantly from those who did not opt in.

## 6. Conclusion

The present study evaluated the reliability and validity of PHQ-9 and GAD-7 among nurses/midwives, and CHVs in Kenya. Our results provide evidence for one-factor structure in PHQ-9 and GAD-7, which is also generalizable across Swahili and English languages and across sex (males and females). Therefore, this study provides a simple, reliable and valid set of tools for national wide usage to screen depression and anxiety among our sample population.

## Data availability statement

The original contributions presented in the study are included in the article/supplementary materials, further inquiries can be directed to the corresponding authors.

## Ethics statement

The studies involving human participants were reviewed and approved by Institutional Scientific and Ethics Review Committee, Aga Khan University. The Ethics Committee waived the requirement of written informed consent for participation.

## Author contributions

AA, CS, and EN-M: conceptualization and methodology. SO, BMN, RO, AA, CS, and EN-M: investigation. RO, PM, and SO: data management. SO, PM, and AA: formal analysis. SO, BMN, AA, CS, and EN-M: project administration and supervision. SO and PM: writing-original draft preparation. SO, PM, BMN, RO, CS, EN-M, and AA: writing-review and editing. All authors read, provided feedback, approved, and agreed to the published version of the manuscript.

## Funding

This work was funded by Johnson and Johnson Foundation [Grant Number 63773339]. The content is solely the responsibility of the authors and does not necessarily represent the official views of Johnson and Johnson Foundation. AA’s work is also supported by the Office of The Director, National Institutes of Health (OD), the National Institute of Biomedical Imaging and Bioengineering (NIBIB), the National Institute of Mental Health (NIMH), and the Fogarty International Center (FIC) of the National Institutes of Health under award number U54TW012089 (AA and AK Waljee).

## Conflict of interest

The authors declare that the research was conducted in the absence of any commercial or financial relationships that could be construed as a potential conflict of interest.

## Publisher’s note

All claims expressed in this article are solely those of the authors and do not necessarily represent those of their affiliated organizations, or those of the publisher, the editors and the reviewers. Any product that may be evaluated in this article, or claim that may be made by its manufacturer, is not guaranteed or endorsed by the publisher.

## Abbreviations

CFA, Confirmatory factor analysis
PSS, Perceived stress scale
CHV, Community health volunteer
PC-PTSD-5, Primary care PTSD screen for DSM-5
COVID, Coronavirus disease
BRS, Brief resilience scale
GAD, Generalized anxiety disorder
OLBI, Oldenburg burnout inventory
FE, Field enumerator
UWES, Utrecht work engagement scale
HIV, Human immunodeficiency virus
KMO, Kaiser-Meyer-Olkin
HRH, Human resources for health
RMSEA, Root mean error of approximation
NCK, Nursing council of Kenya
SRMR, Standardized root mean square residual
PHQ, Patient health questionnaire
CFI, Comparative fit index
NACOSTI, National commission for science, technology and innovation
TLI, Tucker Lewis index
IERC, Institutional ethics research committee
