# Corrigendum: Psychometric evaluation of PHQ-9 and GAD-7 among community health volunteers and nurses/midwives in Kenya following a nation-wide telephonic survey

**DOI:** 10.3389/fpsyt.2023.1253843

**Published:** 2023-08-08

**Authors:** Sabina Adhiambo Odero, Paul Mwangi, Rachel Odhiambo, Brenda Mumbua Nzioka, Constance Shumba, Eunice Ndirangu-Mugo, Amina Abubakar

**Affiliations:** ^1^Institute for Human Development, Aga Khan University, Nairobi, Kenya; ^2^School of Nursing and Midwifery, Aga Khan University, Nairobi, Kenya

**Keywords:** anxiety, depression, community health volunteers, PHQ-9, GAD-7, nurses/midwives, reliability, validity

In the published article, the surname of the fourth author was incorrectly written in the article's citation as “Mumbua Nzioka B” and copyright statement as “Mumbua Nzioka”. It should read “Nzioka BM” and “Nzioka”, respectively.

The author's name was also incorrectly abbreviated in the Author contributions statement as BN, while it should read BMN. This sentence previously stated:

“AA, CS, and EN-M: conceptualization and methodology. SO, BN, RO, AA, CS, and EN-M: investigation. RO, PM, and SO: data management. SO, PM, and AA: formal analysis. SO, BN, AA, CS, and EN-M: project administration and supervision. SO and PM: writing-original draft preparation. SO, PM, BN, RO, CS, EN-M, and AA: writing-review and editing. All authors read, provided feedback, approved, and agreed to the published version of the manuscript.”

The correct statement appears below:

“AA, CS, and EN-M: conceptualization and methodology. SO, BMN, RO, AA, CS, and EN-M: investigation. RO, PM, and SO: data management. SO, PM, and AA: formal analysis. SO, BMN, AA, CS, and EN-M: project administration and supervision. SO and PM: writing-original draft preparation. SO, PM, BMN, RO, CS, EN-M, and AA: writing-review and editing. All authors read, provided feedback, approved, and agreed to the published version of the manuscript.”

In the published article, there was an error. The authors stated that PHQ-9 and GAD-7 were significantly positively correlated with resilience and work engagement instead of negatively correlated in the results section of the abstract.

A correction has been made to Abstract, *Results*, Lines 97–99. This sentence previously stated:

“The PHQ-9 and GAD-7 were also significantly positively correlated with resilience and work engagement, supporting divergent validity.”

The corrected sentence appears below:

“The PHQ-9 and GAD-7 were significantly negatively correlated with resilience and work engagement, supporting divergent validity.”

The authors apologize for this error and state that this does not change the scientific conclusions of the article in any way. The original article has been updated.

